# NOS2 inhibitor 1400W Induces Autophagic Flux and Influences Extracellular Vesicle Profile in Human Glioblastoma U87MG Cell Line

**DOI:** 10.3390/ijms20123010

**Published:** 2019-06-20

**Authors:** Paola Palumbo, Francesca Lombardi, Francesca Rosaria Augello, Ilaria Giusti, Sabino Luzzi, Vincenza Dolo, Maria Grazia Cifone, Benedetta Cinque

**Affiliations:** 1 Department of Life, Health & Environmental Sciences, University of L’Aquila, Building Delta 6, Coppito, 67100 L’Aquila, Italy; paola.palumbo@univaq.it (P.P.); francesca.lombardi@univaq.it (F.L.); francescaaugello@gmail.com (F.R.A.); ilaria.giusti@guest.univaq.it (I.G.); vincenza.dolo@univaq.it (V.D.); mariagrazia.cifone@univaq.it (M.G.C.); 2 Neurosurgery Unit, Department of Clinical-Surgical, Diagnostic and Pediatric Sciences, University of Pavia, Polo Didattico “Cesare Brusotti”, Viale Brambilla, 74 - 27100 Pavia, Italy; sabino.luzzi@unipv.it

**Keywords:** NOS2, glioblastoma, U87MG, glioma stem cells, NOS2 inhibitor 1400W, autophagy, extracellular vesicles

## Abstract

The relevance of nitric oxide synthase 2 (NOS2) as a prognostic factor in Glioblastoma Multiforme (GBM) malignancy is emerging. We analyzed the effect of NOS2 inhibitor 1400W on the autophagic flux and extracellular vesicle (EV) secretion in U87MG glioma cells. The effects of glioma stem cells (GSC)-derived EVs on adherent U87MG were evaluated. Cell proliferation and migration were examined while using Cell Counting Kit-8 assay (CCK-8) and scratch wound healing assay. Cell cycle profile and apoptosis were analyzed by flow cytometry. Autophagy-associated acidic vesicular organelles were detected and quantified by acridine orange staining. The number and size of EVs were assessed by nanoparticle tracking analysis. EV ultrastructure was verified by transmission electron microscopy (TEM). WB was used to analyze protein expression and acid sphingomyelinase was determined through ceramide levels. 1400W induced autophagy and EV secretion in both adherent U87MG and GSCs. EVs secreted by 1400W-treated GSC, but not those from untreated cells, were able to inhibit adherent U87MG cell growth and migration while also inducing a relevant level of autophagy. The hypothesis of NOS2 expression as GBM profile marker or interesting therapeutic target is supported by our findings. Autophagy and EV release following treatment with the NOS2 inhibitor could represent useful elements to better understand the complex biomolecular frame of GBM.

## 1. Introduction

**Figure 1:**
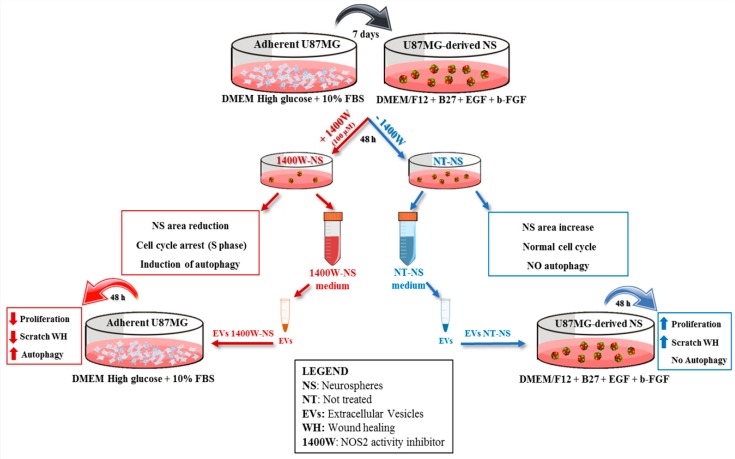
Scheme of experimental design and obtained results.

Glioblastoma Multiforme (GBM), which is the most common glioma, is a lethal primary tumor of the central nervous system, being characterized by a highly infiltrating capacity, striking cellular heterogeneity, relapsing ability, and resistance to therapy. At present, the standard of therapeutic protocol consists of maximum conceivable surgical resection, followed by radiotherapy plus parallel adjuvant chemotherapy with temozolomide (TMZ) [1]. Despite this, resistance to therapy limits its effectiveness and GBM cannot be effectively controlled, being characterized by an extremely wide set of genetic and epigenetic alterations and high rates of recurrences [2,3]. In addition, neuroinflammation also increases after treatment, making the development of alternative therapeutic approaches critically imperative. Indeed, the median patient survival of 15 months, where less than 5% of patients survive for more than five years after diagnosis [4]. Although the complex biomolecular framework that underlies GBM aggressiveness is not yet fully defined, an increasing amount of studies that aimed at identifying the mechanisms responsible for GBM progression, chemo/radiotherapy resistance, and high recurrence rate led to the identification of glioma stem cell (GSC) as an election target for anti-GBM therapy [5,6]. Many of the drugs that were tested to prevent GBM growth and invasiveness are able to kill tumor cells by inducing apoptosis, autophagic cell death or necrosis [7]. Although apoptosis is considered to be the most common form of programmed cell death, there are significant literature data that attribute a noteworthy involvement of autophagic death in the tumorigenesis process [8]. Autophagy is a highly conserved catabolic process, by which cells can recycle organelles and long-lived intracellular proteins [9]. The induction of autophagy can protect or kill metabolically active cancer cells, including GBM cells, depending upon the cellular microenvironment [7,8]. Indeed, autophagy can either sustain cancer cells to evade death in response to stress conditions (i.e., starvation or deprivation of pro-survival signaling, hypoxia, apoptosis, heat or chemical stress, anti-cancer therapies) or support tumor progression as well as a dysregulated or excessive hyperactivation of autophagic flux can lead to non-apoptotic type II programmed cell death, which is known as autophagic cell death. Even with the awareness of its “double face”, autophagy induction has been proposed as another potential anti-tumoral mechanism to counteract several cancers, including GBM [7,8,10]. Recent studies have highlighted the molecular pattern and regulatory pathways that are shared between autophagy and biogenesis of extracellular vesicles (EVs), suggesting that these processes are intimately linked [11]. From some years, our group has focused its attention on the role of the inflammatory process in the GBM biology. In particular, we are interested in investigating the involvement of nitric oxide synthase 2 (NOS2), an inflammation-associated enzyme with oncogenic function in several cancers, including GBM [12,13,14,15]. In this regard, previously, our findings evidenced a significant upregulation of NOS2 in glioma cells grown in specific conditions to promote the GSC expansion as three-dimensional (3D) sphere cultures (neurospheres, NS) [16]. A strong and significant correlation was also found between the expressions of NOS2 and the stemness marker SOX-2 (Sex-determining Region Y-Box 2), which resulted in being upregulated both in GBM cell lines, i.e., U-251MG, T98G, U-87 MG, U-373MG, LN229, and GBM primary cultures [16,17]. Even more recently, the emerging relevance of NOS2 as a prognostic factor in GBM malignancy has been further supported by our results, showing the crucial role of NOS2 expression in proliferation, clonogenic ability, migration, and NS generation, either in U87MG and T98G human glioma cell lines or GBM primary cultures [18]. In the present study, we show evidence that 1400W, one of the most potent and selective NOS2 inhibitor [19,20,21] was able to induce the autophagic flux and EV secretion in adherent U87MG cells, as well as in 3D-cultured GSCs. In addition, the effects of EVs that are released by GSCs previously incubated with or without 1400W have been analyzed on recipient adherent U87MG cells. The results suggest that EVs secreted by 1400W-treated NS, but not those from untreated NS, were able to inhibit U87MG cell growth and migration while inducing a relevant level of autophagy. Figure 1 schematizes the experimental study design together with the main results.

## 2. Results

### 2.1. 1400W Reduces Growth of Glioma Stem Cells by Inducing S-phase Cell Cycle Arrest

**Figure 2:**
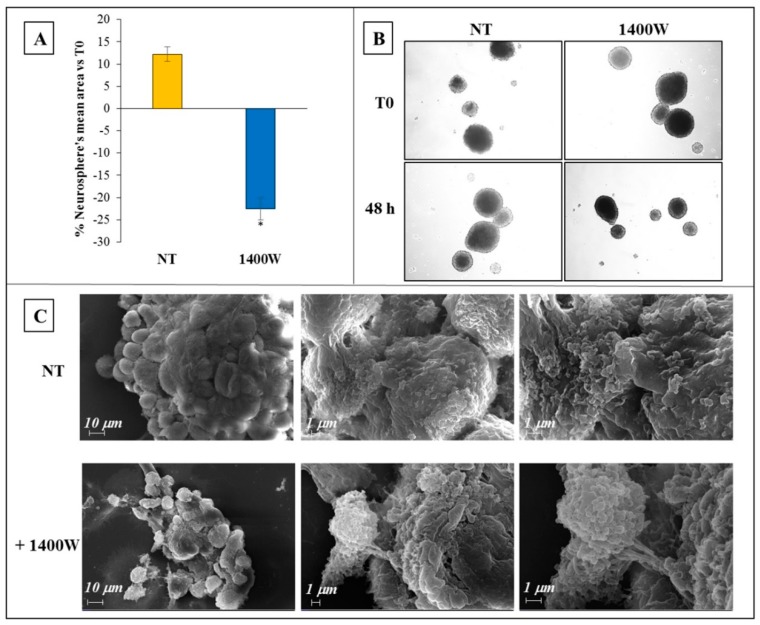
Effect of 1400W on U87MG-derived neurospheres (NS) growth. (**A**) U87MG cells were maintained for 7 days in a specific medium (DMEM/F12+B27+EGF+b-FGF) to allow NS generation afterwich NS were incubated in the absence (not treated, NT) or presence of 1400W (100 μM) for 48 h. Quantitative analysis of NS mean area is expressed as percentage vs. relative T0. The results representative of two independent experiments are expressed as mean values of duplicates ± SD. For comparison between two means, Student’s unpaired *t*-test was used (* *p <* 0.05). (**B**) Representative phase contrast images (4× magnification) of human U87MG-derived NS in the absence (NT) or presence of 1400W (100 μM) for 48 h. (**C**) Representative scanning electron micrograph images of untreated (NT) or 1400W-treated NS (from left to right: 2000×, 10,000×, 20,000× magnification, respectively).

First, the growth of NS previously generated after a seven-day culture of adherent U87MG cells in GSC-M, as described in the Methods’ section, was examined following treatment with 1400W (100 μM) for 48 h. In these conditions, NOS2 inhibitor significantly affected the GSC growth, as assayed by measuring the sphere average area in mm^2^. Indeed, as shown in Figure 2A, while the average surface of untreated NS after 48 h culture was increasing by >10%, the addition of 1400W led to a ~22–23% decrease of NS mean area vs relative T0. The results are expressed as mean ± SD and they are representative of two independent experiments in duplicate. For comparison between means, Student t-test was used (* *p* < 0.05). Representative phase contrast images (4× magnification) of NS not treated or treated with 1400W for 48 h are shown in Figure 2B. With low-magnification (2000×) SEM imaging, the NS surfaces showed more packaged cells in the controls (NT) than in 1400W-treated NS. Moreover, a smaller size of the 1400W-treated NS when compared to controls (NT) was evident, thus confirming above results (Figure 2C, images on the left). Additionally, at high-magnification, the 1400W-treated spheres had a grape-like appearance, with a mostly rough-surface with protrusions and vesicular formations more copious than in NT (Figure 2C, images on the middle (10,000×) and right (20,000×).

**Figure 3:**
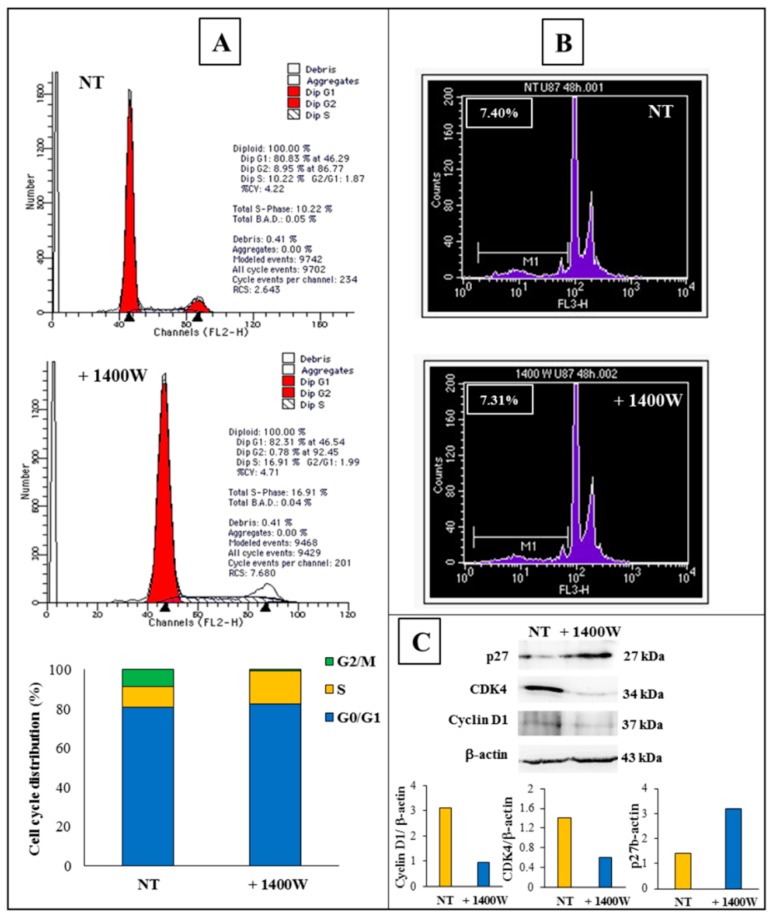
Effects of 1400W on U87MG-derived NS cell cycle. (**A**) Cell cycle profiles of NS-forming cells after 48 h incubation without (NT) or with 1400W (100 μM). Cell cycle distribution (%) is also reported in the histogram. Results are representative of three independent experiments. (**B**) Flow cytometric profiles of apoptosis level analysis in NS-forming cells after 48 h incubation without (NT) or with 100 μM 1400W. Results are representative of three independent experiment. (**C**) Representative images of western blot analysis of cell cycle-related proteins Cyclin D1, CDK4, and p27 in extracts of NS after 48 h incubation without (NT) or with 1400W (100 μM). The results of densitometric analysis of bands expressed as ratio vs. β-actin band intensity are reported in the histograms. Data are representative of two independent experiments.

The inhibition of growth of 3D-cultured GSCs was also supported by the analysis of cellular distribution in cell cycle phases, as assessed by flow cytometry, which revealed that 100 μM 1400W treatment for 48 h was able to induce cell cycle arrest at the S-phase. Indeed, the percentage of cells that were accumulated in the S-phase was noticeably enhanced (16.91%) as compared to the control cells (10.22%). This increase in the S-phase cell population was accompanied by a concomitant decrease in G2/M cell population in the 1400W-treated cells as compared to the control cells (0.78% vs. 8.95%). Figure 3A reports the results that are representative of three independent experiments each performed in duplicate. The data indicate that 1400W mainly reduced GSC proliferation by the induction of cell cycle arrest in the S-phase. On the other hand, 1400W did not induce significant levels of apoptosis in GSC after 48 h of treatment (7.31% vs. 7.40% of not treated cells), as shown by the representative cytofluorimetric profiles in Figure 3B. When considering the increasing evidence clearly showing how specific cell cycle proteins (i.e., Cyclin D1 and cyclin-dependent kinase 4, CDK4) and checkpoints (i.e., cyclin-dependent kinase (CDK)-inhibitor, p27) regulate autophagy [22], we examined the expression levels of cyclin D1 and CDK4 by western blotting. Additionally, the expression level of the cyclin-dependent kinase (CDK)-inhibitor, p27, was assessed as a negative regulatory factor of cell cycle progression. In agreement with the above results on cell cycle distribution, the 1400W treatment effectively induced a down-regulation of the expression of both Cyclin D1 and CDK4, which was associated to an increased expression of p27, thus halting the cells in S-phase (Figure 3C).

### 2.2. 1400W Induces Autophagy of Glioma Stem Cells

**Figure 4:**
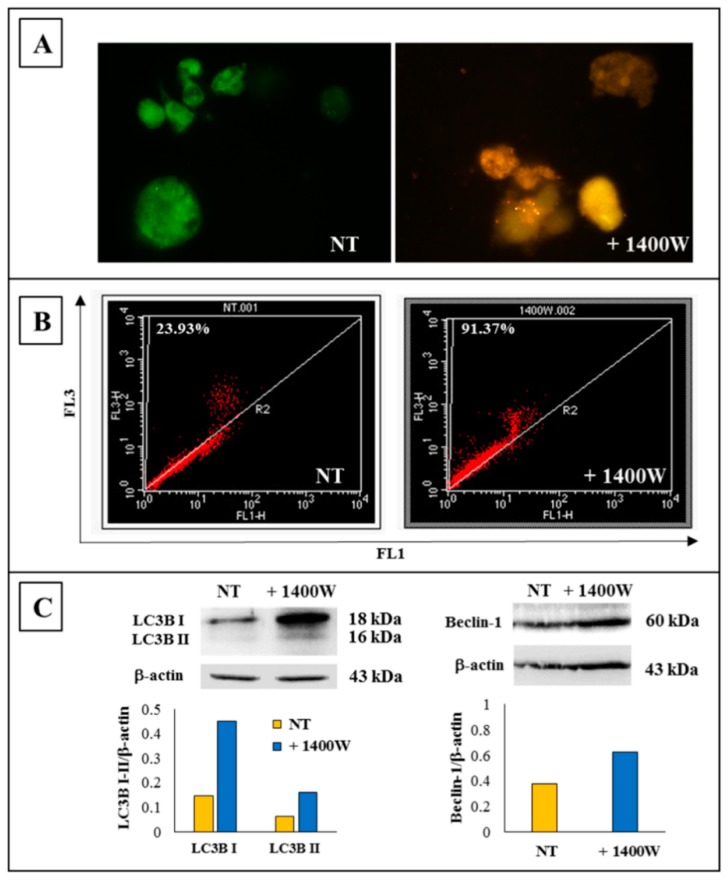
Detection of autophagy by acridine orange staining. (**A**) Representative images of acidic vacuolar organelles (AVO) fluorescence staining in not treated (NT), and 1400W-treated NS-forming cells (100 µM for 48 h) (magnification 100×). (**B**) Flow cytometric analysis of AVO in NS-forming cells exposed or not to 1400W (100 µM) for 48 h. AVO were measured using the 488-nm excitation detector (green fluorescence/FL1) and the 540-nm emission detector (red fluorescence/FL3). The results are representative of two independent experiments. (**C**) Representative images of western blot analysis of autophagic markers LC3B I/II and Beclin-1 in NS treated or not with 1400W (100 µM) for 48 h are shown. The results of densitometric analysis of bands expressed as ratio vs. β-actin band intensity are reported in the histograms. The results are representative of three independent experiments.

The induction of autophagy in NS-forming GSCs after treatment with NOS2 inhibitor 1400W (100 μM) was first revealed through the detection of autophagy-related lysosomal structures, named acidic vesicular organelles (AVO), whose formation is considered as a marker of autophagic process [23]. Acridine orange (AO) staining was used to detect the development of AVO. AO is a metachromatic dye that differentially stains double-stranded and single-stranded nucleic acids. When AO intercalates into double strand DNA, it emits green fluorescence upon excitation at 480–490 nm. On the other hand, AO interacting with single strand DNA or RNA emits red fluorescence, whose intensity is proportional to the degree of acidity. AO staining of the NS-forming cells revealed the accumulation of AVO in the cell cytoplasm after exposure to 100 μM 1400W for 48 h (Figure 4A). Flow cytometry analysis after AO staining was used to quantify the effect of NOS2 inhibitor in terms of the acidity level of AVO. The treatment induced a relevant increase of fluorescence intensity as compared to the untreated cells (91.37% vs. 23.93%) (Figure 4B). Activation of a series of autophagy proteins is considered to be a critical step for autophagosome formation [24]. Accordingly, to monitor the expression of LC3B and Beclin-1, as known key protein markers of autophagic flux [25], western blot analyses were performed on U87MG-derived NS not treated or treated with 1400W for 48 h. In agreement with the above results, the treatment induced an up-modulation of LC3B-I and II and Beclin-1 as compared to the control condition, thus confirming the induction of autophagy. Representative western blots and relative densitometric analyses are shown in Figure 4C.

### 2.3. 1400.W Influences the Release of Extracellular Vesicles by Glioma Stem Cells

**Figure 5:**
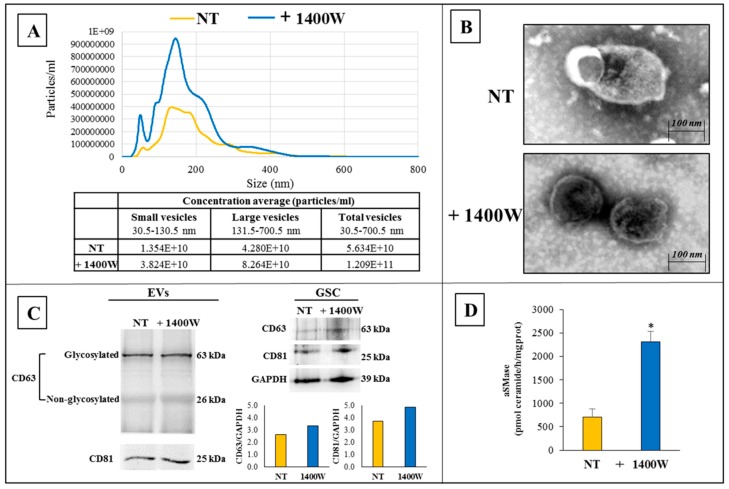
Characterization of extracellular vesicles (EVs) released by U87MG-derived NS after 48 h incubation without or with 1400W. (**A**) Size distribution of EVs from U87MG-derived NS by Nanoparticles Tracking Analysis (NTA). Concentration average of particles per ml is reported in the table. (**B**) Morphological analysis of EVs from U87MG-derived NS, treated or not with 1400W (100 µM) for 48 h, was evaluated using transmission electron microscopy (TEM) (scale bar 100 nm). Pictures show membrane bound vesicles with a spheroid shape. (**C**) Representative western blots of EVs markers, CD63 (core protein, 26 kDa; glycosylated protein, 63 kDa) and CD81, in NS-released EVs and in total NS cellular lysates (NT and 1400W-treated). (**D**) Acid SMase activity in U87MG-derived NS treated or not with 1400W (100 μM) for 48 h. Data are expressed as pmol ceramide/h/mg protein (mean ± SD) and they are representative of four independent experiments in duplicate. For comparison between two means, Student’s unpaired *t*-test was used (* *p <* 0.05).

While taking into account the known association between autophagy and EV biogenesis through shared molecular machinery or organelles [11,26], and also based on the SEM images that showed a significant membrane activity in the NS treated with 1400W (see above, Figure 2C), the ability of NOS2 inhibitor 1400W to influence vesicle secretion by U87MG-derived NS was evaluated. With this aim, we analyzed the EVs that were released by NS cultured with or without 100 μM 1400W for 48 h. The size and concentrations of EVs, as determined by nanoparticle tracking analysis (NTA), are reported in Figure 5A (graph and table). Of note, the total number of EVs (size 30.5–700.5 nm) resulted in being strongly enhanced after treatment with the NOS2 inhibitor, with a % increase >110%. In particular, both small (30.5–130.5 nm) and large (131.5–700.5 nm) vesicle concentrations were increased by 1400W treatment, even if the small vesicle number/mL resulted in being noticeably higher (>180% increase) when compared to large vesicle number/mL (>90% increase). The TEM analysis confirmed the classical morphological features of EVs, as shown by representative images in Figure 5B. The expression of EV protein markers CD81 and CD63 [27] shown in Figure 5C (on the left, representative western blots) also confirmed the nature of EVs. In agreement with the observed increase of EVs that were released by NS treated with 1400W, the expression levels of EV markers CD81 and CD63 were upregulated in treated NS as compared to the not treated ones. Representative western blots and relative densitometric analyses are also shown in Figure 5C (right). Based on the literature data regarding the implication of acid sphingomyelinase (aSMase) in the phenomenon of vesicle biogenesis in response to various stimuli in different cell systems [28], we analyzed the ability 1400W to influence the aSMase activity and the consequent production of ceramide in U87MG-derived NS after treatment with the NOS2 inhibitor for 48 h. The results indicated that 1400W caused a significant enhancement of the level of ceramide that is produced by aSMase, which could be associated with the above reported increase of EV generation that is induced by the NOS2 inhibitor. Figure 5D shows the results that are representative of four independent experiments in duplicate. The enzymatic activity of aSMase is expressed as pmol ceramide/h/mg protein (mean ± SD). It is evident that the addition of 1400W caused an increase of more than 200% of ceramide levels as compared to the untreated control NS (* *p <* 0.05).

### 2.4. EVs Released by 1400W-Treated Glioma Stem Cells Negatively Influence Proliferation and Migration of Adherent U87MG Cells

**Figure 6:**
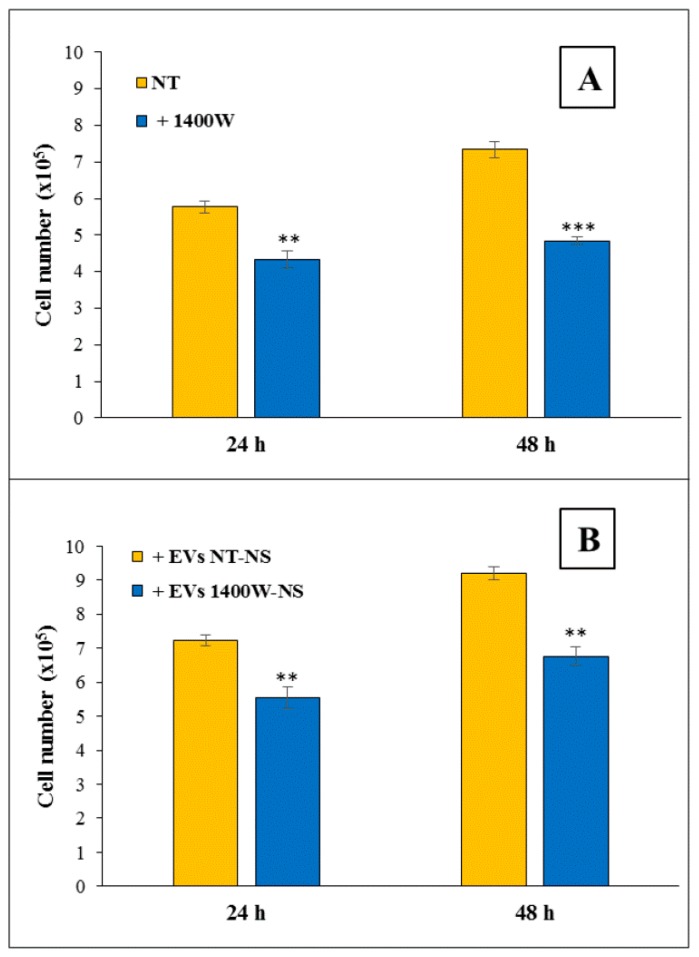
Proliferation assay of adherent U87MG cells treated with 1400W or EVs released by NT-NS or 1400W-treated NS. (**A**) Adherent U87MG cells were treated with 1400W (100 μM) for 24 and 48 h and proliferation rate was measured using Cell Counting Kit-8 assay (CCK-8) assay. Data expressed as total cell number are representative of two experiments in duplicate (** *p <* 0.01, *** *p <* 0.001 compared to relative NT). (**B**) Adherent U87MG cells were incubated with EVs that were released from not treated (EVs NT-NS) and 1400W-treated (EVs 1400W-NS) NS and proliferation assay was assessed at 24 and 48 h. Data that are expressed as total number of cells are representative of two experiments in duplicate (** *p <* 0.01 compared to relative NT).

We first wanted to confirm our previous results about the effects of direct addition of 1400W on U87MG cell proliferation and migration ability to analyze the potential influence exerted by EVs released by U87MG-derived NS on adherent U87MG cells (“recipient cells”) [18]. The results shown in Figure 6 are representative of two independent experiments in duplicate and they are expressed as mean of total viable cell numbers ± SD. As expected, adherent U87MG cell growth evaluated as cell number was significantly reduced in the presence of 100 μM 1400W (** *p <* 0.01 at 24 h; *** *p <* 0.001 at 48 h) (Figure 6A). The % trypan blue stained cells was ever <10% (not shown). 

In agreement with growing literature reports regarding the tumorigenic effects of EVs derived from cancer stem cells in many tumors, including GBM [29], EVs that were released by untreated NS exerted a stimulatory effect (~25% increase) on cell growth of adherent U87MG cells (Figure 6B) when compared to control cells (Figure 6A). Of note, the addition of EVs that were secreted by U87MG-derived NS previously treated for 48 h with 1400W (EVs 1400W-NS) had an impairment effect (~23–28% reduction) on the growth of adherent U87MG cells as compared to that observed in the presence of EVs secreted by NS not treated (EVs NT-NS) (Figure 6B). The percentage range of growth inhibition following the direct addition of 1400W to adherent U87MG cultures appeared to be comparable to that observed following treatment with EVs (Figure 6B), both at 24 h (~20–25%) and at 48 h (~25–35%).

**Figure 7:**
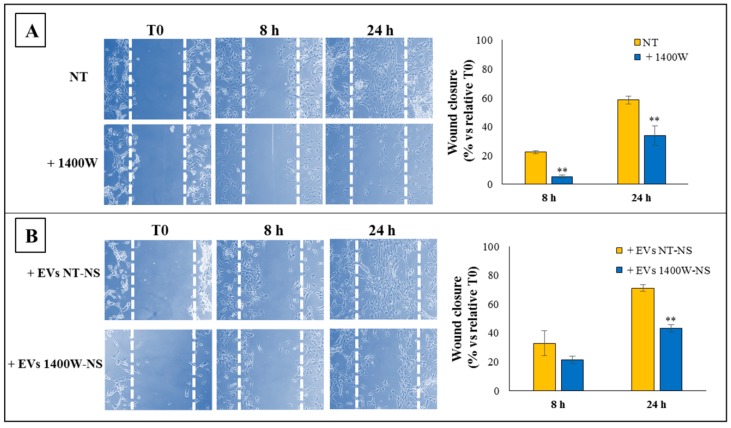
Cell migration ability of adherent U87MG cells treated with 1400W or EVs released by NT-NS or 1400W-treated NS. (**A**) Scratched monolayers of adherent U87MG cells were incubated up to 48 h without (NT) or with 1400W (100 µM) to quantify cell motility by measuring the wound width. Representative microscopy images captured at 0, 8, and 24 h are shown (10× magnification). The quantitative results expressed as % wound closure vs. relative T0 (mean ± SD) are representative from two independent experiments in duplicate. For comparative analysis of groups of data, repeated measures two-way ANOVA followed by Bonferroni post hoc test was used (** *p <* 0.01). (**B**) Wound healing assay performed on adherent U87MG after incubation with EVs derived from untreated NS (EVs NT-NS) and 1400W-treated NS (EVs 1400W-NS). Images of scratched monolayers were acquired for the indicated times after injury (10× magnification). Additionally, in this case, the wound closure rates expressed as % closure at 8 and 24 h vs. relative T0 (mean ± SD) and shown in the histogram are representative of two independent experiments in duplicate. For comparative analysis of groups of data, repeated measures two-way ANOVA, followed by Bonferroni post hoc test was used (** *p <* 0.01).

As tumor invasiveness is one of the pathophysiological features of human GBM, the migration ability of adherent U87MG was also checked through an in vitro scratch wound assay. As yet reported by our group [18], the results confirmed that the ability of the U87MG cells to repair scratched monostrate was strongly and significantly impaired in the presence of the NOS2 inhibitor. Representative microscopy images at T0, 8 h, and 24 h are shown in Figure 7A, together with the relative quantitative analysis of wound-closure rate. The results are expressed as % wound closure vs. relative T0 (mean ± SD) from a representative from two independent experiments in duplicate (** *p <* 0.01). When EVs that were released by U87MG-derived NS previously treated or not with 1400W for 48 h (EVs 1400W-NS) were added to the scratched monolayer of U87MG, the wound closure rate had a pattern that was similar to that observed after the direct addition of 1400W to cell cultures. Indeed, as reported in Figure 7B, the addition of EVs derived from 1400W-treated NS significantly reduced the repair rate of the scratched monolayer at 24 h as compared to the U87MG cells treated with EVs secreted by not treated NS (* *p <* 0.05).

### 2.5. EVs Released by 1400W-Treated Glioma Stem Cells Promote Autophagy of Recipient Adherent U87MG Cells

**Figure 8:**
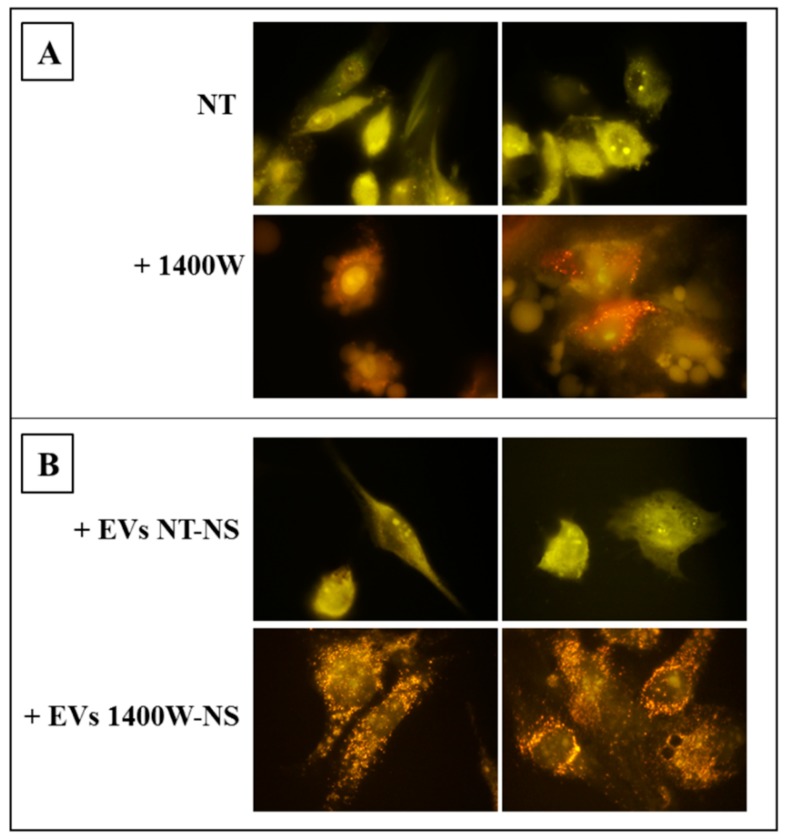
Induction of autophagy by 1400W in adherent U87MG cells. (**A**) U87MG cells were treated or not with 1400W (100 μM) for 48 h and then stained with AO to assess the content of acidic vacuoles (orange/red spots) by fluorescence microscopy. Two images for each condition are shown (magnification 100×) and are representative of two independent experiments. (**B**) U87MG cells were treated for 48 h with EVs derived from NT (EVs NT-NS) and 1400W-treated (EVs 1400W-NS) NS. After treatment cells were stained as above described. Also in this case, two images for each condition are shown and are representative of two independent experiments.

The NOS2 inhibitor 1400W was able to induce high levels of autophagy, even in adherent U87MG cells, as evident in the fluorescence microscope images shown in Figure 8A. Indeed, 1400W-treated cells showed a noticeable increase in red fluorescent AVO, while the control cells (NT) mostly exhibited green fluorescence with no detectable red fluorescence, thus indicating a lack of AVO. In order to evaluate the ability of NS-secreted EVs to also influence autophagy in the recipient cells, the adherent U87MG cells were incubated for 48 h with EVs that were released by NS-forming GSCs previously treated or not with 1400W for 48 h and then stained with AO to verify the development of AVO. As evident from the images shown in Figure 8B, the addition of EVs that were secreted by 1400W-treated NS induced high levels of AVO formation in recipient adherent U87MG cells. On the other hand, EVs from NT-NS did not lead to any formation of AVO.

## 3. Discussion

GBM, being similar to many other malignant tumors, is characterized by a moderately inflammatory microenvironment that promotes all stages of tumorigenesis, tumor progression, and invasiveness, as well as resistance to therapy [30]. In this context, the results of different groups concerning the expression of NOS2, either as a potential GBM molecular profile marker or interesting therapeutic target, assume particular relevance [12,30]. Recently, our group has shown evidence regarding the involvement of NOS2 on human GBM cell growth, clonogenic potential, and neurosphere generation by GSCs [18]. A previous study showed evidence that several NO-releasing chemical compounds were able to decrease endogenous LC3-II in rat primary cortical neurons and HeLa cells, thus suggesting that NO could inhibit autophagy by impairing autophagosome generation [31]. Accordingly, the same authors reported that the inhibition of NO synthesis by L-NAME, which is a broad-spectrum NOS inhibitor, induced autophagy, thus delineating a crucial role of NO in regulating the autophagic process with a number of implications in the context of its multiple cellular functions. Although GBM progression could be prevented by the induction of apoptosis [32], alternative pathways, such as non-apoptotic autophagic cell death, can be just as much or even more effective in causing the death of GBM cells [33]. Of note, cells with a defective non-functional apoptotic pathway may undergo autophagic cell death [34]. Autophagy has been reported to often be triggered before apoptosis, thus making a cell more susceptible to die [35]. However, the complex interplay between autophagy and apoptosis machinery is still controversial and not fully clarified [36]. In particular, the prognosis of GBM could be influenced by autophagy, either positively [37,38] or negatively [39,40,41,42], as well as a defective autophagic pathway has been associated with GBM [43]. Of note, autophagy may limit the tumor-associated inflammation profile through the removal of inflammasomes as well as damaged mitochondria, which are considered to be crucial in supporting the inflammatory microenvironment determinant for tumor progression and invasiveness [44]. Autophagy activation has also been associated with the impairment of GBM cell migration and invasion, which could conversely be stimulated by autophagy inhibition [45]. In this respect, it is noteworthy that the apoptotic pathway is often mutated in human tumors, including GBM [8,36]. Autophagy can then represent a valid alternative form of programmed cell death to prevent tumor growth and progression. 

Based on these findings and with the aim of highlighting the functional role of NOS2 in GBM biology, in this study we explored the possible involvement of the autophagic process and associated vesicle biogenesis in the in vitro anti-tumoral actions of a selective NOS2 inhibitor, 1400W on U87MG cell line, which basically expresses high levels of NOS2 [17]. In our experimental conditions, the NOS2 inhibitor was able to cause greater induction of autophagy markers, including a relevant number of autophagy vacuoles and the upregulation of proteins that are associated to autophagosome generation i.e., LCB3 and Beclin-1, where overexpression in the U87MG cells has been already related to cellular autophagy [46]. Our findings also suggest that the autophagic flux that is induced by 1400W was associated with membrane blebbing, cell cycle arrest, and decreased cell proliferation and migration rate. We also show evidence that 1400W-induced autophagy was accompanied by a remarkable increase of the EV secretion by GSCs, as well as by the upregulation of common protein markers of autophagosome biogenesis (i.e., CD63 and CD81). In addition, the activity of the acidic SMase, an enzyme that is involved in the budding of EV by converting sphingomyelin into ceramide [28,47], was significantly increased in NS-forming GSCs after the addition of 1400W. 

Of note, the results of the experiments designed to explore the effects of EVs released by glioma stem cells previously incubated with 1400W on adherent U87MG cells showed a strong negative influence on the recipient cells, with a severely reduced proliferation rate and migration ability. These effects were associated with a relevant autophagy. Conversely, the adherent U87MG cells receiving EVs secreted by untreated GSCs were induced to an increase of proliferation index and migration ability. No autophagy was detectable in this condition. Although our results indicated that the autophagic flux induced by 1400W was associated with cell cycle arrest without detectable apoptosis, reduced proliferation level, as well as a compromised migration capacity, further experiments are needed to test whether the effect that is induced by the NOS2 inhibitor effectively causes autophagic cell death. It will be also interesting to explore the potential influence of 1400W on PI3K-Akt/mTOR system, a key signaling pathway mainly involved in modulating autophagy [48,49] and is associated to malignancy grade of gliomas [50] in our experimental system. In this respect it is worth noting that the U87MG cell line is expressing mutant PTEN (phosphatase and tensin homolog), which is an important negative regulator of the Akt/mTOR pathway [33].

Taken together, our findings lead us to hypothesize that the effect that is induced by the direct addition of 1400W to glioma stem cells or adherent U87MG cells could be mediated by anti-tumoral molecular messages induced by NOS2 inhibition in the origin cells and then transferred to the surrounding recipient cells through the EVs. On the other hand, NOS2 inhibition could induce GSCs to release EVs with a modified cargo that is able to alter and manipulate the tumor inflammatory microenvironment in order to make it less advantageous to tumor growth and invasiveness.

## 4. Materials and Methods

### 4.1. U87MG Cell line, Glioma Stem Cells, and Treatments

U87MG human grade IV glioma cell line was purchased from the European Collection of Authenticated Cell Cultures (ECACC, Salisbury, UK). The cells were maintained in DMEM High glucose (Dulbecco’s Modified Eagle Medium, EuroClone, West York, UK) supplemented with 10% (v/v) of fetal bovine serum (FBS), 2 mM L-glutamine, 100 U/mL penicillin, and 100 mg/mL streptomycin (named standard medium), and then incubated in sterile conditions at 37 °C in a 5% CO_2_ humidified atmosphere. The cells grow adherent in this culture condition. After reaching 80% confluence, the adherent cell cultures were expanded; sub-culturing was performed every three days. Cell count was evaluated in a Bürker chamber by optical microscopy (Eclipse 50i, Nikon Corporation, Kawasaki, Kanagawa, Japan) while using trypan blue solution (0.04%, final concentration, Euro Clone, West York, UK). In order to obtain the NS cultures, 5 × 10^5^ U87MG cells were cultured in a specific medium composed by DMEM/F12 (1:1, vol/vol) serum free, 20 ng/mL of both recombinant human epidermal growth factor (EGF) and fibroblast growth factor basic (b-FGF) (both acquired from ImmunoTools, 26169, Gladiolenweg 2, Friesoythe, Germany), B27 supplement (Life Technology Corporation, Carlsbad, CA, USA), penicillin/streptomycin and glutamine, as previously reported [16]. Subsequently, the cells were incubated at 37 °C in a humidified atmosphere with 5% CO_2_ in sterile conditions. Where not otherwise specified, the reagents for cell biology and consumables were purchased from EuroClone (EuroClone, West York, UK). After seven days from the addition of glioma stem cell-specific medium (GSC-M), the NS were treated or not with the selective NOS2 inhibitor, N-(3-(aminomethyl)benzyl)acetamidine (1400W) (Sigma-Aldrich, Saint Louis, MO, USA) at the concentration of 100 µM for 48 h. The immunophenotype of GSCs was routinely evaluated by flow cytometry for common stemness marker expression, as previously described [17]. In particular, 3D cultured-GSCs showed high positivity for β-tubulin (>60%), nestin (>80%), and SOX-2 (>60%) (data not shown). The selective NOS2 inhibitor was stored as stock solutions at −20 °C and was diluted in cell culture medium just before use. For each experiment, the NOS2 gene and protein expression levels were preliminarily verified by RT-PCR and western blot, respectively, either in adherent U87MG cells or 3D cultured-GSCs, as described [16,18]. Similarly, NOS2 activity measured through nitrite levels was also routinely checked to verify the efficacy of 1400W as an enzyme inhibitor. NS morphology was detected and analyzed by microscope Nikon Eclipse TS100 at initial time (T0) and after 48 h from treatment. The NS area was assessed at the beginning and at the end of treatment in the absence or presence of 1400W (100 µM). Briefly, 10 bright field images at 4× magnification were randomly taken from not treated and 1400W-treated NS and analyzed while using Image J software. The NS average area (total area/number of NS) was expressed in mm^2^.

### 4.2. Scanning Electron Microscopy

Scanning electron microscopy (SEM) was carried out on U87MG-derived NS that was previously untreated or treated with 1400W and then left to adhere overnight on coverslips that were pre-coated with poly-lysine (1 × 10^3^ cells/cm^2^) and fixed with 2% glutaraldehyde (Electron Microscopy Sciences, Hatfield, PA, USA) in PBS for 30 min. The coverslips were briefly rinsed with PBS and water and then dehydrated in ethanol solutions 30–50–70–90% in H_2_O and three times 100%, for 10 min. each. For HMDS drying, the samples were immersed for 3 min. in 100% HMDS (Electron Microscopy Sciences, Hatfield, PA, USA) and then the excess HMDS was blotted away by filter paper. The samples were then transferred to a desiccator for 25 min. to avoid water contamination, mounted on stubs, sputter-coated with chromium in a Quorumtech Q 150T ES Turbo chromium sputter, and detected via a Zeiss Gemini SEM 500.

### 4.3. Cell Cycle Profile and Apoptosis Analysis by Flow Cytometer

The cell cycle and apoptosis analysis were carried out by cell DNA staining with propidium iodide (PI). U87MG-derived NS, untreated and treated with 1400W at 100 µM for 48 h, were enzymatically dissociated by Accutase™ solution from PAA-GE Healthcare Life Sciences (GE Healthcare Bio-Sciences AB, SE-751 84 Uppsala Sweden) for about 10 min. at 37 °C and then centrifuged (800× *g* for 10 min at 4 °C). The resulting pellets were washed in PBS and fixed in ice-cold ethanol (70%) at 4 °C for 30 min. The fixed cells were transferred to plastic BD tubes (Becton Dickinson, San José, CA, USA), washed twice with the ice-cold PBS, and stained with a mixture solution of PI (50 μg/mL), Nonidet-P40 (0.1% v/v), and RNase A (6 μg/10^6^ cells) (all from Sigma-Aldrich, Saint Louis, MO, USA) for 1 h in the dark at 4 °C. Cell cycle phase distribution was analyzed while using FACSCalibur flow cytometry that was equipped with the cell cycle analysis software Modfit LT for Mac V3.0 (Beckton Dickinson, San Jose, CA, USA). Data from 10,000 events per sample were collected and analyzed. The apoptotic cells were determined by their hypochromic subdiploid nuclei staining profiles and analyzed while using Cell Quest software program (BD Instruments Inc., San José, CA, USA). 

### 4.4. Detection and Quantification of Acidic Vesicular Organelles (AVO) by Fluorescent Staining with Acridine Orange

The adherent cells were seeded on coverslips, treated as described in Results’ section, and then a vital staining with acridine orange (AO) was performed. On the other hand, after treatment, NS were disaggregated by Accutase™, seeded on coverslips, treated as described above, and then performed vital staining with AO. Briefly, both the adherent cells and NS-forming cells were stained with a final concentration of 0.01 µg/mL AO solution in PBS at room temperature for 10 min. and in the presence of RNase A (6 μg/10^6^ cell, Sigma Aldrich). Afterwards, the cells were washed with PBS and examined under a fluorescent microscope (Nikon Eclipse 50i) that was equipped with a digital camera. The presence of orange/red stained cells is due to the accumulation of acidic vacuolar organelles (AVO) in a cytoplasmic compartment. To quantify the development of AVO, the adherent cells (not treated or treated with 100 μM 1400W) were stained, removed from the plate with trypsin-EDTA, and analyzed using a FACSCalibur flow cytometry that was equipped by Cell Quest software. Disrupted NS were counted, stained and analyzed by flow cytometry. Cells were analyzed using the 488-nm excitation detector (green fluorescence/FL1) and the 540-nm emission detector (red fluorescence/FL3), which quantified the development of AVO. The U87MG cells subjected to serum withdrawal for 4 h have been used for the positive control of autophagy.

### 4.5. Isolation and Characterization of NS-Derived Extracellular Vesicles

The EVs were isolated from U87MG-derived NS cell media by ultracentrifugation, as described [51]. Briefly, all of the supernatants from NS cultures exposed or not to 1400W for 48 h were initially cleared of cellular debris/dead cells by centrifugation at 600× *g* for 10 min. (to remove suspended cells), and then at 1500× *g* for 30 min. (to remove cell debris). The collected supernatants were centrifuged at 100,000× *g* (Rotor 70Ti, Quick-Seal Ultra-Clear tubes, k_adj_ 221, brake 9) for 2 h in an Optima XPN-110 Ultracentrifuge (Beckman Coulter, Brea, CA, USA). All of the centrifugations were performed at 4 °C. The pelleted EVs were resuspended in PBS. The quantity of EVs was double measured by determining the total protein concentration in the preparations using the BCA Protein Assay Kit (Pierce, Rockford, IL, USA), following the manufacturer’s instructions. The samples were immediately used or stored at −20 °C. Identification of purified EVs was achieved by morphological examination while using a transmission electron microscope.

### 4.6. Nanoparticle Tracking Analysis (NTA)

The number and dimension of EVs that were released by U87MGNS were assessed by the nanoparticle tracking analysis (NTA). EVs were visualized by laser light scattering using a NanoSight NS300 system (NanoSight Ltd., Amesbury, Wiltshire, UK) equipped with a Blue488 laser and a digital camera sCMOS. Briefly, the EV-enriched pellets (derived from an equal volume of conditioned medium collected from cells originally seeded in the same number) were resuspended in 500 μL of 0.1 μm triple-filtered sterile PBS and five recordings of 30 sec were performed for each sample. The camera level and detection threshold were set at values of 13 and 5, respectively. The collected data were analyzed with NTA 3.3 Dev Build 3.3.301 software, which provided high-resolution particle size distribution profiles and concentration measurements of the vesicles in solution. 

### 4.7. Transmission Electron Microscopy

Transmission electron microscopy (TEM) was performed on isolated EVs and resuspended in PBS to analyze their ultrastructural morphology. According to proper dilutions, the samples were adsorbed to 300 mesh carbon-coated copper grids (Electron Microscopy Sciences) for 5 min. in a humidified chamber at room temperature. The EVs on grids were then fixed in 2% glutaraldehyde (Electron Microscopy Sciences) in PBS for 10 min. and then briefly rinsed in Milli-Qwater. The grids with adhered EVs were examined with a Zeiss Gemini SEM 500 equipped with a STEM detector at 20 kV and at a 3.0 mm working distance, after negative staining with 2% phosphotungstic acid, brought to pH 7.0 with NaOH [51].

### 4.8. Western Blot Analysis

Western blot technique has been performed on lysates of untreated and 1400W-treated NS and their relative EVs to allow for the detection of autophagy related proteins (microtubule-associated protein light chain 3-LC3B and Beclin-1), and members of the tetraspanin protein family, CD63 and CD81, widely used as EVs markers [27]. Moreover, the protein expression of known cell cycle regulators, such as Cyclin D1, CDK4, and p27, has been verified. U87MG derived-NS were digested with RIPA buffer (phosphate buffer saline pH 7.4), supplemented with 0.5% sodium deoxycholate, 1% NP40, 0.1% SDS, 5 mM of EDTA (ethylenediaminetetraacetic acid), 100 mM of protease inhibitor cocktail (Sigma-Aldrich, Milan, Italy). For LC3B and Beclin-1 detection, 25 μg/lane of U87MG-derived NS extracts were run on 12.5% SDS polyacrylamide gels under reducing conditions with β-mercaptoethanol 5%, and the proteins were transferred onto nitrocellulose membranes. For CD63 and CD81 detection, an equal amount of EV proteins (10 μg/lane) were resolved by 10% SDS polyacrylamide gels under reducing conditions with β-mercaptoethanol 5%, and the proteins were transferred onto nitrocellulose membranes. The non-specific binding sites were blocked with 5% non-fat dry milk for 1 h at room temperature, and the membranes were incubated overnight at 4 °C with primary antibodies. All of the primary antibodies are listed here: anti-LC3B (Thermo Fisher, Waltham, MA, USA), anti-Beclin-1 (Origene, 9620 Medical Center Drive Suite 200 Rockville, MD, USA), anti-CD63, and anti-CD81 were acquired from Novus Biological (Centennial, CO, USA), anti-Cyclin D1, anti-CDK4, and anti-p27 (Sigma-Aldrich, Saint Louis, MO, USA). The bands were visualized by ECL chemiluminescent substrate reagent, according to the manufacturer instructions and acquired by UVItec Alliance (Cambridge, UK). Densitometric analysis was performed by software that was provided by the company. The protein band intensities were normalized to relative β-actin or GAPDH band. 

### 4.9. Acid Sphingomyelinase Activity

After incubation with or without 100 μM 1400W for 48 h, U87MG-derived NS were lysed in 250 mM sodium acetate (pH 5.0) and 1% NP40 for 30 min. in ice. The samples were then subjected to centrifugation at 10,000× *g* at 4 °C for 10 min. The supernatant was isolated and a BCA protein assay kit measured the protein concentration (Pierce, Rockford, IL, USA). Acid sphingomyelinase (aSMase) enzymatic activity was assayed according to the previously reported method [52]. For each sample, an aliquot corresponding to 30 µg of proteins was diluted in a buffer containing 250 mM sodium acetate, 1 mM EDTA, pH 5.0. The enzyme reaction was started by the addition of 10 nmol of C12-NBD Sphingomyelin (N-[12-[(7-nitro-2-1,3-benzoxadiazol-4-yl) amino]dodecanoyl]-sphingosine-1-phosphocholine Avanti Polar Lipids, Inc, Alabaster, Alabama) in acid reaction buffer (250 mM sodium acetate, 1 mM EDTA and 0.2% Triton-X100, pH 5.0) in a total volume of 200 µL. After incubation at 37 °C for 1 h, the reaction was stopped by the addition of 200 µL chloroform:methanol (2:1, v/v), the samples were centrifuged for 10 min. at 22,000× *g*, the organic phases were extracted, and, at the aqueous phases, 400 µL chloroform:methanol (2:1, v/v) were added. The samples were centrifuged and the organic phases were added to the organic lipid phases previously obtained. The organic lipid phases were evaporated under a N_2_ stream and then dissolved in 80 µL chloroform. The samples were spotted onto a thin-layer chromatography (TLC) plate (Merck, Kenilworth, NJ, USA) and separated with chloroform:methanol:water (65:25:4, v/v/v) being used as a solvent. Under these conditions, NBD-ceramide appeared as a single spot. The emission intensities of the fluorescent ceramide spots were determined by UVItec Alliance (Cambridge, UK). Densitometric analysis was performed by software that was provided by the company. The amounts of pmol ceramide generated from NBD-SM by aSMase activity were obtained from interpoling the respective fluorescence intensities in the calibration plot of C12-NBD Ceramide (N-[12-[(7-nitro-2-1,3-benzoxadiazol-4-yl)amino]dodecanoyl]-D-erythro-sphingosine, Avanti Polar Lipids, Inc, Alabaster, Alabama) concentration vs. fluorescence intensity. The aSMase activity was expressed as picomoles ceramide produced/h/mg protein.

### 4.10. Cell Proliferation Assay

Cell proliferation was examined while using Cell Counting Kit-8 assay (CCK-8, Sigma-Aldrich, Saint Louis, MO, USA), according to the manufacturer’s instructions. Briefly, the U87MG cells were seeded into 96-well plates at a density of 3 × 10^3^ cells per well in 100 μL culture medium, incubated overnight, then treated or not with 100 µM 1400W for 24 and 48 h. Where indicated, the cells were incubated for 24 and 48 h with EVs that were derived from not treated (EVs NT-NS) or 1400W-treated (EVs 1400W-NS) NS for 48 h. 10 μL CCK-8 reagent was added to each well and incubated at 37 °C for 2 h. The absorbance was recorded at a wavelength of 450 nm while using a microplate reader (Bio-Rad, Hercules, CA, USA). A calibration curve has been prepared using the data that were obtained from the wells that contain known numbers of viable cells.

### 4.11. Scratch Wound Healing Assays

A scratch assay was performed, as previously described, to evaluate the effect of 1400W or EVs secreted by untreated or 1400W-treated GSCs on proliferation and migration of adherent U87MG cell line [53]. Briefly, the cells were plated at 6 × 10^4^/cm^2^ in multiwell plates and cultured until reaching confluence. DMEM was removed and the cell monolayers were scratched while using a 200 µL pipet tip. Subsequently, the cells were washed with PBS and cultured with fresh medium at 37 °C in a 5% CO_2_ humidified atmosphere in the absence or presence of 100 µM 1400W or EVs (30 µg/mL) collected from not treated (EVs NT-NS) and 1400W treated NS (EVs 1400W-NS). The images of cell migration were taken by the inverted light microscope (Eclipse TS 100, Nikon) at 10× magnification at different time points after the injury (0, 8, 24 h). The experiments were conducted in duplicate, and six fields for each condition were analyzed. The images were analyzed quantitatively while using the standalone TScratch software that automatically calculates the portion of area that is occupied by the cells by a mathematical model to calculate the percentage of wound closure.

### 4.12. Statistical Analysis

Statistical analysis of data was performed while using GraphPad Prism 6.0 (GraphPad Software, San Diego, CA, USA). Student’s unpaired *t*-test was used for comparison between two means. For comparison of the mean values among the groups, a two-way ANOVA, followed by Bonferroni post hoc test, were used. The results were expressed as mean ± SD, as specified in figure legends. *p* values less than 0.05 were considered to be statistically significant.

## Abbreviations

NOS2	Nitric Oxide Synthase 2
GBM	Glioblastoma Multiforme
1400W	N-(3-(Aminomethyl)benzyl)acetamidine
GSCs	Glioma Stem Cells
EVs	Extracellular vesicles
NS	Neurospheres
CDK4	Cyclin dependent kinase 4
AVO	Acidic vesicular organelles
AO	Acridine orange
GSC-M	Glioma stem cell-specific medium
GAPDH	Glyceraldehyde 3-phosphate dehydrogenase
